# The Prevalence and Implications of Polypharmacy in Individuals With Type 1 Diabetes

**DOI:** 10.1002/cpt.70130

**Published:** 2025-11-12

**Authors:** Namam Ali, Stephan van Erp, Cornelis Kramers, Cornelis J. Tack, Bastiaan E. de Galan

**Affiliations:** ^1^ Department of Internal Medicine Radboud University Medical Center Nijmegen The Netherlands; ^2^ Department of Pharmacy Radboud University Medical Center Nijmegen The Netherlands; ^3^ Department of Internal Medicine Maastricht University Medical Center+ Maastricht The Netherlands; ^4^ CARIM School for Cardiovascular Disease Maastricht University Maastricht The Netherlands

## Abstract

Polypharmacy is increasingly recognized as a relevant issue in diabetes care, but its prevalence and clinical relevance in individuals with type 1 diabetes remain underexplored. This study aimed to determine the prevalence of polypharmacy and to identify associated clinical and psychological factors. Participants were recruited from a tertiary diabetes outpatient clinic between February 2020 and April 2021. Polypharmacy was defined as the concurrent use of five or more medications, including insulin. Clinical, sensor‐based, and psychosocial data were collected. Logistic regression was used to identify variables independently associated with polypharmacy. A total of 484 individuals with type 1 diabetes were included (mean age 51.3 ± 15.9 years; 51.2% male; median diabetes duration 30 [IQR 16–40] years; mean HbA_1c_ 60.3 ± 11.6 mmol/mol). Polypharmacy was present in 175 (36.2%) participants. Individuals with polypharmacy were more often female, were older, and had longer diabetes duration, higher BMI, higher HbA_1c_, more complications, and higher rates of hospital admission. They also were more likely to have impaired awareness of hypoglycemia and reported higher levels of fear of hypoglycemia with no differences in hyperglycemia‐related worry or behavior or diabetes‐related emotional distress. Polypharmacy affects over one‐third of individuals with type 1 diabetes and is associated with poorer health status and a greater hypoglycemia‐related burden. Future studies should investigate whether targeted medication review and psychological interventions may alleviate some of the burden in this high‐risk group.

**Study Highlights**

**WHAT IS THE CURRENT KNOWLEDGE ON THE TOPIC?**

Polypharmacy is highly prevalent in type 2 diabetes due to comorbid disease and complications. Individuals with type 1 diabetes are also at high risk of comorbidities, often requiring pharmacological interventions. Few data exist on the prevalence of polypharmacy and associated clinical and psychological factors in people with type 1 diabetes.

**WHAT QUESTION DID THIS STUDY ADDRESS?**

What is the prevalence of polypharmacy in a contemporary cohort of individuals with type 1 diabetes? What are the associations between polypharmacy and clinical factors, including continuous glucose monitoring (CGM) data, as well as psychological factors?

**WHAT DOES THIS STUDY ADD TO OUR KNOWLEDGE?**

The prevalence of polypharmacy among individuals with type 1 diabetes is 36% in a tertiary medical center. Polypharmacy is associated with older age, longer diabetes duration, higher HbA_1c_, and more complications. Individuals with polypharmacy more often have impaired awareness of hypoglycemia and report significantly higher fear of hypoglycemia.

**HOW MIGHT THIS CHANGE CLINICAL PHARMACOLOGY OR TRANSLATIONAL SCIENCE?**

Polypharmacy is a prevalent and clinically relevant issue in type 1 diabetes requiring routine assessment. Future studies should examine whether targeted medication reviews and psychological interventions can alleviate some of the burden of polypharmacy in people with type 1 diabetes.

Recent estimates indicate that approximately 8.4 million individuals worldwide are currently living with type 1 diabetes, with the prevalence expected to increase significantly, reaching 13.5–17.4 million by 2040.^1^ As healthcare advancements continue to improve life expectancy, individuals with type 1 diabetes are not only living longer but also are also facing an increasing risk of diabetes‐related complications and cardiovascular diseases.^2^, ^3^ In addition to insulin therapy, managing long‐term complications and associated cardiovascular risks often necessitates additional pharmacological intervention. Also, the psychological impact of living with type 1 diabetes and the complexity of its management sometimes require interventions that may include pharmacotherapy.

Pharmacological therapies play an important role in both the prevention and management of diabetes‐related complications and cardiovascular diseases. The World Health Organization defines polypharmacy as “the administration of many drugs at the same time or the administration of an excessive number of drugs”.^4^ Although no universally accepted threshold exists, polypharmacy is commonly defined operationally as the concurrent use of five or more drugs.^4^, ^5^ While prescribing multiple medications is often clinically necessary, its implementation can lead to adverse outcomes, particularly when medications are no longer indicated or result in side effects, including those arising from drug–drug interactions. The risks associated with polypharmacy are magnified by the unique challenges faced by individuals with chronic conditions like type 1 diabetes, including adverse drug reactions, medication non‐adherence, drug–drug interactions, heightened hospitalization rates, and increased mortality.^5^, ^6^ For individuals with type 1 diabetes, these risks are further amplified by the additional complications associated with polypharmacy, such as an increased likelihood of falls, hypoglycemia, and diabetic ketoacidosis, with each additional medication potentially compounding these risks.^7^

The global prevalence of polypharmacy in the general population is estimated at 37% (95% confidence interval (CI): 31–43%).^5^ Among individuals with type 2 diabetes, polypharmacy is even more prevalent, affecting 54% in primary care settings and up to 82% in academic healthcare settings.^8^ The exact prevalence of polypharmacy in individuals with type 1 diabetes remains underexplored. A single population‐based register study reported polypharmacy to range from 29% among individuals aged 40–49 years to 76% among those aged 80 years and over.^7^ Also, little is known about clinical and psychological factors associated with polypharmacy in individuals with type 1 diabetes. The primary objective of this study is therefore to assess the prevalence of polypharmacy in a cohort of individuals with type 1 diabetes from an academic center in the Netherlands and to investigate its associations with clinical factors, including continuous glucose monitoring (CGM) data, and psychological factors, including diabetes‐related distress.

## MATERIALS AND METHODS

### Study design and population

This was a post hoc analysis of a single‐center, cross‐sectional cohort study.^9^ Participants with type 1 diabetes were recruited from the diabetes outpatient clinic of the Radboud University Medical Centre from February 2020 through April 2021. Inclusion criteria for the study were a clinical diagnosis of type 1 diabetes, age ≥ 16 years, sufficient comprehension of the Dutch language and the ability to provide informed consent as judged by the investigator. Participants were excluded when the medical history mentioned severe psychiatric comorbidity or other comorbidity interfering with the completion of the questionnaires or the provision of informed consent. Severe psychiatric comorbidity was defined as a psychiatric disorder resulting in serious functional impairment, which substantially interferes with or limits one or more major life activities. Also, individuals with a (recent, i.e., <3 months) hospital admission for any psychiatric disorder were excluded from participating. Participants who consented to participate in the study were invited to the outpatient clinic of our hospital or – when in‐person visits were not feasible a virtual appointment was made for data collection.

### Ethics

Ethical approval for the study was obtained from the Institutional Review Board of the Radboud University Medical Centre (NL‐71207.091.19) and the study was conducted according to the principles expressed in the Declaration of Helsinki. All participants gave written informed consent before participation.

### Demographics and clinical characteristics

Clinical characteristics, including age, gender, diabetes duration, mode of insulin treatment, mode of glucose monitoring, insulin dose, self‐reported frequency of hypoglycemic events per week and any severe events in the past year, presence of micro‐ and macrovascular diabetes complications, smoking, alcohol use and medication use were obtained by questionnaires and verified against clinical records whenever possible. BMI was measured at the outpatient clinic or retrieved from the clinic record if the appointment for data collection was virtual. Furthermore, blood was drawn to measure glycated hemoglobin A_1c_ (HbA_1c_) or this was derived from the patient record if such a measurement had been done no longer than 3 months earlier. Hypoglycemia awareness status was assessed with the Clarke questionnaire, where a score of four or more out of seven classifies for impaired awareness of hypoglycemia (IAH).^10^ A score less than four was regarded as normal awareness of hypoglycemia (NAH). Severe hypoglycemia was defined in accordance with the definition of the American Diabetes Association Working Group on Hypoglycemia, as an event requiring assistance of another person to actively administer carbohydrate, glucagon or other resuscitative actions, in the last year (i.e., the year before entering the study).^11^

### Polypharmacy

Polypharmacy was defined as the concurrent use of five or more drugs.^4^, ^5^ For the purpose of this study, we further categorized polypharmacy into three distinct levels: no polypharmacy (1–4 drugs), moderate polypharmacy (5–9 drugs) and severe polypharmacy (10 or more drugs). Insulin use was included in the polypharmacy count. The number of medications was defined as the number of concomitantly used, unique medications. Fixed dose combinations were counted by their number of active pharmaceutical ingredients. Glucagon was excluded, as its use is typically temporary and restricted to acute episodes of hypoglycemia. Similarly, antibiotics were excluded as these were used by fewer than 10 participants and only for short periods of infections (typically <10 days). Pro re nata (PRN) medications were included in the count if they were used more than three times per week. Information on the use of oral contraceptives was not collected and was therefore not considered in the polypharmacy assessment.

Medications were categorized into distinct therapeutic classes for analysis. Antihypertensive drugs were subdivided into beta‐blockers, renin‐angiotensin‐aldosterone system inhibitors (including angiotensin‐converting enzyme inhibitors and angiotensin receptor blockers), diuretics, aldosterone antagonists, calcium channel blockers and vasodilatory drugs (i.e., alpha‐blockers and nitrates). Antithrombotic therapy included platelet aggregation inhibitors, direct oral anticoagulants and vitamin K antagonists. Psychotropic drugs included antipsychotic, antidepressive, antiepileptic and benzodiazepines. Corticosteroids were included regardless of their route (oral, nasal, topical, inhaled). Immunosuppressive and immunomodulatory drugs included all types of immunosuppressive drugs other than corticosteroids. Analgesics were defined as acetaminophen, non‐steroidal‐anti‐inflammatory drugs and opiates. Drugs related to acid disorders included all acid lowering therapies used for gastric protection. Anti‐osteoporosis drugs, aimed at improving bone strength, were defined as agents that either slow bone resorption or stimulated bone formation, including calcium, vitamin D, bisphosphonates, RANK ligand inhibitors and parathyroid hormone analogues. Drugs related to endocrine disorders are drugs used for thyroidal diseases and other non‐thyroidal endocrinopathies. Ophthalmologicals were defined as medications targeting ocular conditions and drugs for obstructive airway disease were defined as medications targeting asthma or COPD, excluding corticosteroids. Urologicals were classified as those addressing bladder and prostate conditions, predominantly alpha‐1‐receptors. Vitamins and mineral supplements are included vitamin D, vitamin B12, calcium, zinc, and other over‐the‐counter micronutrient preparations. A separate category, “other drugs” were drugs that did not fit into any of the aforementioned groups, such as erythropoietin, pancreatic enzyme supplements and glucosamine.

### Intermittently scanned continuous glucose monitoring

Data from is‐CGM were collected for the participants who were using is‐CGM from online data sharing platforms. is‐CGM data (representation of 28 days) used for the analysis were: time sensor is active (% of time), average number of scans per day, average glucose, estimated HbA_1c_, glucose variability, time below target range (% of time glucose ≤3.9 mmol/l), time in target range (% of time glucose 3.9–10.0 mmol/L), time above target range (% of time glucose >10.0 mmol/l), number of hypoglycemic events (glucose ≤3.9 mmol/l) and duration of such hypoglycemic events.

### Diabetes distress questionnaires

The Problem Areas in Diabetes (PAID‐5) survey was used to measure diabetes‐related emotional distress.^12^ This questionnaire consists of five questions with response on a five‐point Likert scale, ranging from 0 (not a problem) to 4 (serious problem), with total scores ranging from 0–20. A total score of 8 or more indicates elevated diabetes‐related emotional distress.

Fear of hypoglycemia was assessed with the 33‐item Hypoglycaemia Fear Survey‐II (HFS‐II) questionnaire,^13^ which consists of two subscales, one for behavior (HFS‐B, items 1–15) and the other for worry (HFS‐W, items 16–33). HFS‐B describes behaviors in which individuals try to avoid hypoglycemic episodes and/or their negative consequences, whereas HFS‐W items describe specific concerns that individuals with diabetes may have about their hypoglycemic episodes. Responses are given on a five‐point Likert scale, ranging from 0 (never) to 4 (almost always). Total and subscale scores are reported as the sum of the item rankings, with maximum scores of 60, 72 and 132 for HFS‐B, HFS‐W, and HFS‐II total, respectively. Higher scores indicate greater fear of hypoglycemia.

The Hyperglycaemia Avoidance Scale (HAS) survey was used to evaluate measures taken to prevent hyperglycemia (10 questions) and the concerns and feelings about hyperglycemia (11 questions).^14^ Participants rated their level of agreement with statements on a Likert scale, ranging from 0 (never) to 4 (always). Higher scores indicate greater worry and avoidance behavior related to hyperglycemia.

### Statistics

Continuous data are expressed as mean (± standard deviation) if normally distributed or median [interquartile range] if not normally distributed. Categorical data are presented as number (percentage, %). Demographics and clinical characteristics, is‐CGM data, diabetes distress questionnaires and drugs were compared between participants with and without polypharmacy using independent Student *t*‐test or Mann–Whitney U‐test for continuous variables, depending on their distribution, and χ^2^ test was used for categorical data.

We examined the demographic and clinical characteristics and diabetes distress questionnaires associated with polypharmacy using both univariable and multivariable logistic regression analyses. For the multivariable analysis, we included the significant variables identified in the univariable analysis and constructed three distinct models to assess their relationships with the risk of polypharmacy. In model 1 of the analysis, we adjusted for age, gender and BMI; model was additionally adjusted for HbA_1c_ and IAH; and model 3 was additionally adjusted for microvascular complications, macrovascular complications, alcohol use, hospitalization in past year and HFS‐II total score. Odds ratio (OR) with 95% CIs are reported for the logistic regression analysis.

To evaluate the association between age and polypharmacy, participants were categorized into predefined age groups, and a Chi‐square test with a linear‐by‐linear association was performed. Exploratively, age was dichotomized at successive cut‐offs (X = 30, 40, …, 80 years), and each pair of groups (<X vs. ≥X) was compared using a Chi‐square test. The lowest age cutoff for which the Chi‐square test yielded *P* < 0.05 was considered the age at which polypharmacy became significantly different.

A *P* value of <0.05 was considered statistically significant. All statistical analyses were performed with IBM SPSS version 25 software (Armonk, NY). Figures were made with Microsoft Office Excel 2024.

## RESULTS

Of the 701 individuals with type 1 diabetes initially identified as potentially eligible for inclusion 217 individuals were excluded for various reasons, as described previously^9^ (**Figure** **S1**).

**Table** **1** summarizes the demographic and clinical characteristics of the study cohort. The mean age of participants was approximately 51 years, with a balanced distribution across gender. The average diabetes duration was 30 years, and overall glycemic control was satisfactory. Continuous glucose monitoring was widely adopted, with 85% of participants using either is‐CGM or rt‐CGM systems. The average reported numbers of hypoglycemic events per week and severe hypoglycemic events in the past year were 3 and 1, respectively. Microvascular complications were present in 55% of participants, while macrovascular complications were documented in 14%.

**Table 1:** Clinical characteristics

	Total group (*n* = 484)	No polypharmacy (*n* = 309)	Polypharmacy (*n* = 175)
Age, years	51 ± 16	45 ± 14	62 ± 13**
Males, *n* (%)	249 (51.4)	174 (56.3)	75 (42.9)**
BMI, kg/m^2^	25.8 ± 4.2	25.3 ± 3.8	26.8 ± 4.7**
HbA_1c_, mmol/mol (%)	60.3 ± 11.6 (7.7 ± 1.1)	58.4 ± 11.0 (7.5 ± 1.0)	63.8 ± 11.8 (8.0 ± 1.1)**
Diabetes duration, years	30 [16–40]	24 [14–36]	37 [27–48]**
CSII, *n* (%)	243 (50.2)	148 (47.9)	95 (54.3)
Insulin dose, EH/kg/day	0.6 [0.5–0.7]	0.6 [0.5–0.7]	0.6 [0.4–0.8]
Mode of glucose monitoring, *n* (%)			
SMBG	72 (14.9)	42 (13.6)	30 (17.1)
is‐CGM	356 (73.6)	234 (75.7)	122 (69.7)
rt‐CGM	39 (8.1)	21 (6.8)	1 (10.3)
Number of hypoglycemic events, *n*/week	3 [2–5]	3 [2–5]	3 [2–6]
Number of severe hypoglycemic events in past year, *n*/patient‐year	1 [0–2]	1 [0–2]	1 [1–3]
IAH (Clarke), *n* (%)^a^	89 (18.4)	44 (14.2)	45 (25.7)**
Microvascular complications, *n* (%)			
Total	266 (55.0)	135 (43.7)	131 (74.9)**
Retinopathy	216 (44.6)	109 (35.3)	107 (61.1)**
Nephropathy	60 (12.4)	22 (7.1)	38 (21.7)**
Neuropathy	151 (31.2)	59 (19.1)	92 (52.6)**
Macrovascular complications, *n* (%)			
Total	66 (13.6)	10 (3.2)	56 (32.0)***
Ischemic heart disease	38 (7.9)	2 (0.6)	36 (20.6)**
Cerebrovascular disease	24 (5.0)	8 (2.6)	146 (9.2)**
Peripheral arterial disease	20 (4.1)	4 (1.3)	16 (9.1)**
Smoking, *n* (%)	60 (12.4)	39 (12.6)	21 (12.0)
Alcohol use, *n* (%)	366 (75.6)	246 (79.6)	120 (68.6)**
Hospitalization in past year, *n* (%)	26 (5.4)	11 (3.6)	15 (8.6)*

CSII, continuous subcutaneous insulin infusion; HbA_1c_, glycated hemoglobin A_1c_; IAH, impaired awareness of hypoglycemia; is‐CGM, Intermittently scanned continuous glucose monitoring; NAH, normal awareness of hypoglycemia; rt‐CGM, real time‐continuous glucose monitoring; SMBG, self‐monitoring of blood glucose.

Data are presented as mean ± standard deviation, median [IQR] or number (%) as appropriate.

*P < 0.05, **P < 0.01: vs. no polypharmacy.

^a^ A score of ≥4 out of seven classifying for IAH and a lower score regarded as normal awareness of hypoglycemia (NAH).^10^

The prevalence of polypharmacy in the current study population was 36.2% (*n* = 175) with a median number of prescribed drugs of 3.^2^, ^3^, ^4^, ^5^, ^6^ Among those with polypharmacy, 78.3% were taking 5–9 drugs, while 21.7% were using 10 or more. All medication classes were more often used among individuals with polypharmacy compared to those without (**Table** **S1**). The most prevalent medication subgroups were antihypertensive drugs (43.2%), particularly renin‐angiotensin inhibitors (34.1%), and lipid modifying drugs (34.7%). See **Table** **S1** for details.

**Figure 1:**
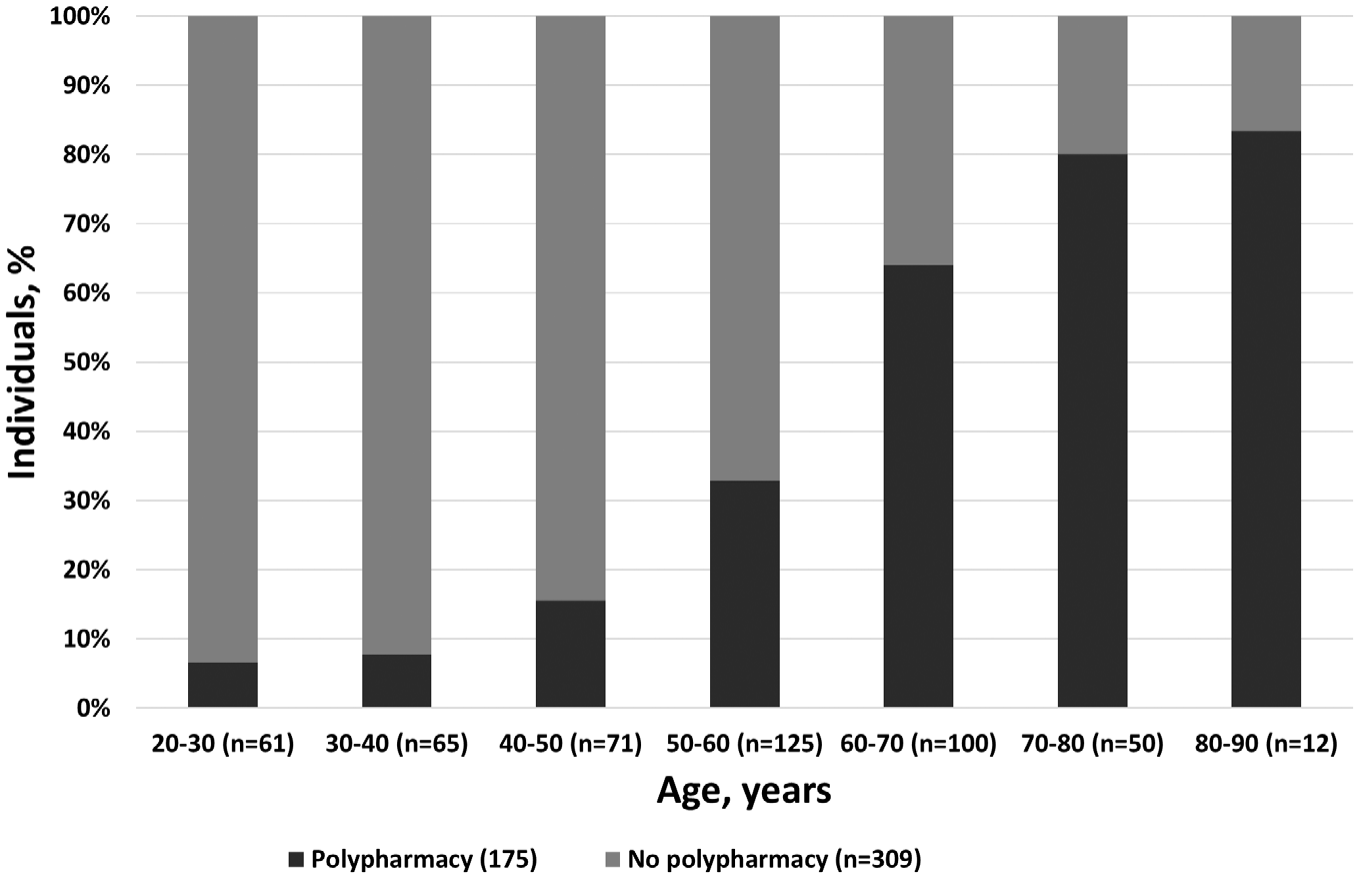
Age distribution in people with and without polypharmacy. Chi‐square test and linear‐by‐linear association showed a significant increase from age ≥ 40 (*P* < 0.05).

Compared to those without polypharmacy, individuals with polypharmacy were significantly older, were more often female, had higher BMI, higher HbA_1c_ and longer diabetes duration, and more frequently had microvascular and macrovascular complications (**Table** **1**). Additionally, they reported less alcohol consumption and were more likely to have been hospitalized in the past year. There were no differences between the groups with respect to insulin pump use, insulin dose, and history of (severe) hypoglycemic events, although those with polypharmacy more often had IAH. The mode of glucose monitoring system did not significantly differ between individuals with and without polypharmacy, although numerically individuals in the polypharmacy group used SMBG and rt‐CGM more often than is‐CGM. The proportion of individuals with polypharmacy increased with age, ranging from 11.5% for participants aged between 20 and 50 years up to 82.9% in those aged between 50 and 80 years (**Figure** **1**). In people over 60 years, the majority was characterized by polypharmacy (*P* < 0.001). An explorative Chi‐square test showed that individuals aged 40 years and older had significantly higher rates of polypharmacy compared to those under 40 years.

Is‐CGM data were available for 292 (60%) individuals, 32% of whom with polypharmacy (**Table** **2**). Participants with or without polypharmacy used their sensor equally often with 85% using it more than 70% of the time. Except for slightly lower TBR among individuals with polypharmacy, there were no differences between the groups regarding time in range, time above range, glucose variability, hypoglycemic events and estimated HbA_1c_. The discrepancy between laboratory‐measured and sensor‐estimated HbA_1c_ could not be explained by differences between individuals with and without sensor use (**Table** **S2**).

**Table 2:** Intermittently scanned continuous glucose monitoring data related to polypharmacy

	No polypharmacy (*n* = 198)	Polypharmacy (*n* = 94)
Time sensor is active, %	87.0 ± 20.8	86.4 ± 23.1
Scan frequency per day, *n*	10 [6–15]	10 [6–14]
Time in target range (3.9–10.0 mmol/l), %	59.3 ± 16.9	57.9 ± 15.7
Time below target range, %		
<3.9 mmol/l	3.7 ± 3.5	2.8 ± 2.6*
<3.0 mmol/l	0.7 ± 1.5	0.6 ± 1.7
Time above target range, %		
>10.0 mmol/l	24.3 ± 8.5	26.3 ± 8.9
>13.9 mmol/l	12.1 ± 12.6	12.4 ± 10.5
Average glucose, mmol/l	9.2 ± 1.8	9.5 ± 1.6
Glucose variability, %	36.7 ± 6.3	36.0 ± 5.8
Estimated HbA_1c_, mmol/mol (%)	55.4 ± 9.7 (7.3 ± 0.8)	56.9 ± 7.5 (7.3 ± 0.7)
Hypoglycemic events (glucose < 3.9 mmol/l), *n*	12 [5–26]	11 [4–17]
Duration of hypoglycemic events (glucose < 3.9 mmol/l), minutes	87 [65–116]	81 [61–105]

Data are presented as mean ± standard deviation or median [IQR] as appropriate.

* *P* < 0.05 vs. no polypharmacy group.

Individuals with polypharmacy had significantly higher scores on the fear of hypoglycemia subscales for both behavior and worry subscale, indicating greater concern and avoidance behavior related to hypoglycemia. No significant differences were observed in the Diabetes‐Related Emotional Distress or Hyperglycaemia Avoidance subscales (**Table** **3**).

**Table 3:** Polypharmacy related to diabetic distress

	No polypharmacy (*n* = 299)	Polypharmacy (*n* = 123)
A. PAID‐5 (diabetes‐related emotional distress)		
PAID‐5 ≥ 8, *n* (%)	59 (19.1)	43 (24.6)
Total score	5.0 ± 4.0	5.3 ± 4.1
B. HFS‐II (fear of hypoglycemia)		
Total score	27.8 ± 16.9	34.5 ± 20.9**
HFS‐B subscale score	14.4 ± 7.3	18.2 ± 9.9**
HFS‐W subscale score	13.4 ± 11.5	16.4 ± 12.7*
E. HAS (hyperglycemia avoidance)		
Total score	33.0 ± 10.2	33.6 ± 10.0
HAS behavior to avoid hyperglycemia subscale	18.2 ± 4.6	18.6 ± 4.9
HAS emotions around hyperglycemia subscale	14.8 ± 7.4	15.0 ± 6.9

HAS, hyperglycemia avoidance survey; HFS‐II, hypoglycemia fear survey‐II; PAID‐5, Problem Areas in Diabetes survey.

*P < 0.05, **P < 0.01 vs. no polypharmacy.

In the univariable logistic regression model, several factors were significantly associated with polypharmacy, including age, female gender, BMI, HbA_1c_, diabetes duration, IAH, microvascular complications and macrovascular complications, alcohol abstinence, hospitalization in the past year and HFS‐II score (**Table** **S3**). In the multivariable regression model, after adjusting for all covariates included in model 3, age, BMI, HbA_1c_, presence of macrovascular complications and absence of alcohol use remained independently associated with polypharmacy (**Table** **4**).

**Table 4:** Multivariable logistic regression analysis relating clinical parameters to polypharmacy

	Model 1 OR (95% CI)	Model 2 OR (95% CI)	Model 3 OR (95% CI)
Age	1.10 (1.08–1.12)^a^	1.10 (1.07–1.12)^a^	1.08 (1.05–1.10)^a^
Gender^b^	0.52 (0.33–0.82)^a^	0.56 (0.35–0.90)^a^	0.60 (0.35–1.02)
BMI	1.07 (1.02–1.13)^a^	1.06 (1.00–1.21)^a^	1.06 (1.01–1.12)^a^
HbA_1c_		1.04 (1.02–1.07)^a^	1.02 (1.01–1.05)^a^
IAH		1.74 (0.98–3.12)	1.43 (0.80–2.54)
Microvascular complications			1.43 (0.80–2.54)
Macrovascular complications			4.36 (1.87–10.19)^a^
Alcohol use			0.54 (0.27–0.94)^a^
Hospitalization in past year			2.91 (0.77–10.91)
HFS‐II total score			1.00 (0.99–1.02)

95% CI: 95% confidence interval; HbA_1c_, glycated hemoglobin A_1c_; HFS‐II: hypoglycemia fear survey‐II; IAH, impaired awareness of hypoglycemia.

Model 1: age, gender^#^, BMI. Model 2: age, gender, BMI, HbA_1c_, IAH. Model 3: age, gender, BMI, HbA_1c_, IAH, microvascular complications, macrovascular complications, alcohol use, hospitalization in past year, HFS‐II total score.

^a^ Association being statistically significant.

^b^ Reference: male.

## DISCUSSION

This study estimated the prevalence of polypharmacy among individuals with type 1 diabetes in our center at ~36%. Our findings further demonstrate that individuals with polypharmacy were more often female, older, had a longer diabetes duration, higher BMI, higher HbA_1c_ levels, were more often affected by IAH and diabetes‐related complications, and had higher fear of hypoglycemia. After adjustment for baseline characteristics, we found that higher age, BMI, and HbA_1c_, presence of macrovascular complications, and abstinence from alcohol were independently associated with polypharmacy. Despite the difference in HbA_1c_, however, there were no differences regarding CGM recorded glucometrics, including time in different ranges and glucose variability.

The prevalence of polypharmacy observed in our study (36%) is broadly comparable to a previous study that reported an average prevalence of 27.2% (excluding insulin) in people with type 1 diabetes and an about similar increase with increasing age.^7^ Our data are also in line with a Scottish population‐based study, in which the mean number of prescribed drugs in people with type 1 diabetes was 4.00 ± 4.35.^7^ Studies in mixed (type 1 and type 2) diabetes populations from the Netherlands and Brazil reported a higher prevalence of polypharmacy of 58%^15^ and 57%,^16^ respectively, while a study in Saudi Arabia reported an even higher prevalence of 78%.^17^ This may be explained by the generally much higher prevalence of polypharmacy in type 2 diabetes populations,^8^ in whom the more extensive use of cardiovascular risk management therapies reflects the higher burden of cardiovascular disease in this group. Nevertheless, the risk factors associated with polypharmacy that we identified in our study, including older age, longer diabetes duration, higher BMI, and presence of diabetes‐related complications, are in line with previous findings obtained in those with type 2 diabetes.^5^, ^7^, ^8^, ^16^, ^17^, ^18^, ^19^

There were significant associations between polypharmacy and both higher HbA_1c_ levels and higher prevalence of IAH, suggesting more fluctuations in glycemic control. The association between polypharmacy and IAH may be partly explained by polypharmacy’s impact on physiological and cognitive processes. The use of multiple medications, including those for comorbidities, can alter glucose metabolism and mask hypoglycemic symptoms (e.g., non‐selective beta‐blockers).^20^ Additionally, the complex regimens often inherent in polypharmacy may increase treatment burden and cognitive load that potentially may interfere with timely recognition and management of hypoglycemia. This complexity may also negatively affect medication adherence, further contributing to suboptimal glycemic control. Individuals with polypharmacy also expressed higher levels of fear of hypoglycemia, perhaps as a consequence of IAH. Fear of hypoglycemia is well documented to negatively impact self‐management and insulin titration. This may lead some patients to adopt higher glucose levels than recommended.^21^, ^22^ This may call upon the HCP to prescribe additional medication to optimize glucose management or because of complications associated with poor glucose control. These observations extend current knowledge by suggesting that polypharmacy in type 1 diabetes is not only a marker of cumulative chronic disease burden, but may also be associated with poorer self‐management outcomes and greater psychological distress.

We found no significant differences between groups in concerns about hyperglycemia or broader diabetes‐related distress. This suggests that the psychological burden associated with polypharmacy is specifically related to hypoglycemia. As such, clinicians need to assess and address hypoglycemia‐related anxiety directly, particularly in individuals managing complex medication regimens. These findings underscore that polypharmacy in type 1 diabetes is a clinical signal that should prompt more than medication review alone. It requires an integrated approach involving psychological support, individualized glycemic targets, and proactive management of hypoglycemia risk. Future studies should explore interventions tailored to this subgroup, with the goal of improving not only glycemic outcomes but also treatment satisfaction and quality of life.

In individuals with polypharmacy, HbA_1c_ levels were higher compared to those without polypharmacy, while CGM‐derived HbA_1c_ estimates (i.e., GMI), time‐in‐range, time‐above‐range, and glycemic variability in those with a sensor did not differ between the groups. CGM‐derived GMI and laboratory‐measured HbA_1c_ often show clinically relevant discrepancies, highlighting that these measures may reflect different aspects of glycemic control.^23^ These observations align with existing literature indicating that fear of hypoglycemia and therapeutic conservatism can influence glycemic targets and insulin dosing.^24^

A strength of this analysis lies in the use of a comprehensive, multidimensional dataset that combines clinical and demographic data with sensor‐based metrics and validated assessments of mental health. This integrative approach provides a more nuanced understanding of the complex relationships between medication burden, glycemic control, and psychological well‐being—elements rarely examined simultaneously in prior studies.

Several limitations must also be acknowledged. First, the observational cohort design precludes causal inference. Second, the recruitment from a single academic healthcare setting may limit the generalizability of our results to less specialized centers, although this may be less so for the associations between polypharmacy and clinical parameters. Nonetheless, the cohort was demographically representative of a broader population of individuals with type 1 diabetes. Third, the relatively short period of CGM monitoring may not fully reflect long‐term patterns of glycemic variability or hypoglycemic episodes. To what extent this explains the discrepancy between measured HbA_1c_ and CGM requires further with longer follow‐up. Fourth, we did not collect data about the history of psychological disorders, such as anxiety and depression. However, we expect these numbers to be low given the infrequent use of psychiatric mediation (27.5%), a large proportion of which may have been provided for painful neuropathy. Finally, psychosocial data were self‐reported, introducing potential reporting bias. To reduce this risk, we used well‐validated and widely accepted questionnaires with established reliability and validity in similar populations.

In summary, polypharmacy affects over a third of individuals with type 1 diabetes and is associated with a distinct and clinically relevant profile, including older age, longer diabetes duration, higher BMI, poorer glycemic control, and more diabetes‐related complications. Notably, polypharmacy is associated with a higher prevalence of IAH and increased fear of hypoglycemia. These findings underscore the importance of a holistic and multidisciplinary approach to managing polypharmacy in individuals with type 1 diabetes, that may include regular medication reviews and providing support to reduce fear and improve awareness of hypoglycemia. Future research should prioritize longitudinal and interventional studies to elucidate causal mechanisms, evaluate the impact of deprescribing strategies, and develop personalized interventions aimed at reducing medication burden while improving both metabolic control and psychological well‐being in this high‐risk population.

## FUNDING

CT received funding from the Perspectief Biomarker Development Center Research Programme, which is (partly) financed by the Netherlands Organisation for Scientific Research (NWO). A number of authors (NA, BDG, CT) were part of the European research project HypoRESOLVE (Hypoglycaemia – Redefining SOLutions for better liVEs).

## CONFLICT OF INTEREST

The authors declare no competing interests for this work.

## AUTHOR CONTRIBUTIONS

NA and BDG designed the research; NA performed the research; NA and SE analyzed the data. NA, BDG, CK, and CT wrote the manuscript.

## ETHICS STATEMENT

Ethical approval for the study was obtained from the Institutional Review Board of the Radboud University Medical Centre (NL‐71207.091.19).

## Supporting information


Data S1.

## Data Availability

The datasets generated during and/or analyzed during the current study are available from the corresponding author on reasonable request.
