# Supplementary material for: The Prevalence and Implications of Polypharmacy in Individuals With Type 1 Diabetes
Source: Clin Pharmacol Ther. 2025 Nov 12;119(3):696–702. doi: 10.1002/cpt.70130 (PMC12882754; doi:10.1002/cpt.70130)

**SUPPLEMENTARY**

**Table S1: Drug use**

|  | | **No polypharmacy (n= 309)** | **Polypharmacy (n= 175)** |
| --- | --- | --- | --- |
| **Blood pressure lowering drugs** | Total | 62 (20.1%) | 147 (84.0%)^*^ |
|  | Beta-blockers | 5 (1.6%) | 67 (38.3%)^*^ |
|  | Renin-angiotensin inhibitors | 51 (16.5%) | 114 (65.1%)^*^ |
|  | Aldosterone antagonists | 0 (0%) | 6 (3.4%)^*^ |
|  | Calcium channel blockers | 10 (3.2%) | 46 (26.3%)^*^ |
|  | Diuretics | 11 (3.6%) | 69 (39.4%)^*^ |
|  | Vasodilators | 3 (1.0%) | 23 (13.1%)^*^ |
| **Antithrombotic therapy** | | 13 (4.2%) | 81 (46.3%)^*^ |
| **Lipid modifying agents** | | 55 (17.8%) | 113 (64.6%)^*^ |
| **Psychotropic drugs** | | 18 (5.8%) | 38 (21.7%)^*^ |
| **Anti-histaminics** | | 10 (3.2%) | 25 (14.3%)^*^ |
| **Corticosteroids** | | 16 (5.2%) | 46 (26.3%)^*^ |
| **Immunosuppressive/modulatory therapy other than corticosteroids** | | 5 (1.6%) | 20 (11.4%)^*^ |
| **Analgesics** | | 8 (2.6%) | 40 (22.9%)^*^ |
| **Drugs related to acid disorders** | | 13 (4.2%) | 75 (42.9%)^*^ |
| **Anti-emetics** | | 1 (0.3%) | 7 (4.0%)^*^ |
| **Constipation therapy** | | 5 (1.6%) | 27 (15.4%)^*^ |
| **Anti-osteoporotic therapy** | | 3 (1.0%) | 39 (22.3%)^*^ |
| **Drugs related to endocrine disorders** | | 54 (17.5%) | 48 (27.4%)^*^ |
| **Ophthalmologicals** | | 13 (4.2%) | 39 (22.3%)^*^ |
| **Urologicals** | | 1 (0.3%) | 9 (5.1%)^*^ |
| **Drugs for obstructive airway disease** | | 3 (1.0%) | 21 (12.0%)^*^ |
| **Vitamins and mineral supplements** | | 46 (14.9%) | 95 (54.3%)^*^ |
| **Other** | | 29 (9.4%) | 57 (32.6%)^*^ |

*p<0.01 vs no polypharmacy

**Table S2: Laboratory-measured and CGM-estimated HbA_1c_ in individuals with and without polypharmacy, stratified by sensor data availability**

|  | **With sensor data** | | |  | **Without sensor data** | | |
| --- | --- | --- | --- | --- | --- | --- | --- |
| **Measured HbA_1c,_ mmol/mol (%)** | **Total (n=282)** | **No polypharmacy (n=191)** | **With polypharmacy (n=91)** |  | **Total (n=165)** | **No polypharmacy (n=95)** | **With polypharmacy (n=70)** |
|  | 60.1±11.1 (7.7±1.1) | 58.5±10.6 (7.5±1.0) | 63.5±11.5 (8.0±1.1)^*^ |  | 60.7±12.3 (7.7±1.1) | 58.1±11.7 (7.5±1.1) | 64.3±12.2 (8.0±1.1)^*^ |
| **CGM-estimated HbA_1c_, mmol/mol (%)** | 55.8±9.1 (7.3±0.7) | 55.3±9.7 (7.3±0.8) | 56.9±7.5 (7.4±0.7) |  | **-** | **-** | **-** |

*p<0.01: vs no polypharmacy

Abbreviations: CGM, continuous glucose monitoring; HbA_1c_, glycated haemoglobin A_1c_

**Table S3: Univariable logistic regression analysis relating clinical parameters to polypharmacy**

| **Demographics** | | **OR (95% CI)** |
| --- | --- | --- |
| Age | | 1.10 (1.08-1.12) |
| Male | | 0.58 (0.40-0.85) |
| BMI | | 1.09 (1.04-1.14) |
| HbA_1c_ | | 1.04 (1.02-1.06) |
| Diabetes duration | | 1.05 (1.04-1.07) |
| CSII | | 1.28 (0.89-1.86) |
| Daily insulin dose | | 0.99 (0.87-1.10) |
| Mode of glucose monitoring (reference: SMBG) | FGM | 0.73 (0.44-1.22) |
|  | rt-CGM | 1.20 (0.55-2.63) |
| Number of hypoglycaemic events in week | | 0.99 (0.94-1.05) |
| Number of severe hypoglycaemic events in past year | | 1.01 (0.94-1.09) |
| IAH (Clarke score ≥4) | | 2.09 (1.31-3.32) |
| Microvascular complications | | 3.93 (2.60-5.93) |
| Macrovascular complications | | 14.3 (7.06-28.99) |
| Smoking | | 0.93 (0.53-1.64) |
| Alcohol | | 0.53 (0.34-0.81) |
| Hospitalization in past year | | 2.56 (1.15-5.70) |
| PAID-5 ≥8 | | 1.43 (0.91-2.26) |
| HFS-II total score | | 1.02 (1.01-1.03) |
| HAS total score | | 1.01 (0.99-1.03) |

Abbreviations: 95% CI: 95% confidence interval; CSII, continuous subcutaneous insulin infusion; is-CGM, Intermittently scanned continuous glucose monitoring; HbA_1c_, glycated haemoglobin A_1c_; IAH, impaired awareness of hypoglycaemia; HAS: hyperglycaemia avoidance survey; HFS-II: hypoglycaemia fear survey-II; OR: odds ratio; rt-CGM, real time-continuous glucose monitoring; PAID-5: Problem Areas in Diabetes survey; SMBG: self-monitoring of blood glucose

**Figure S1: Study flow diagram**


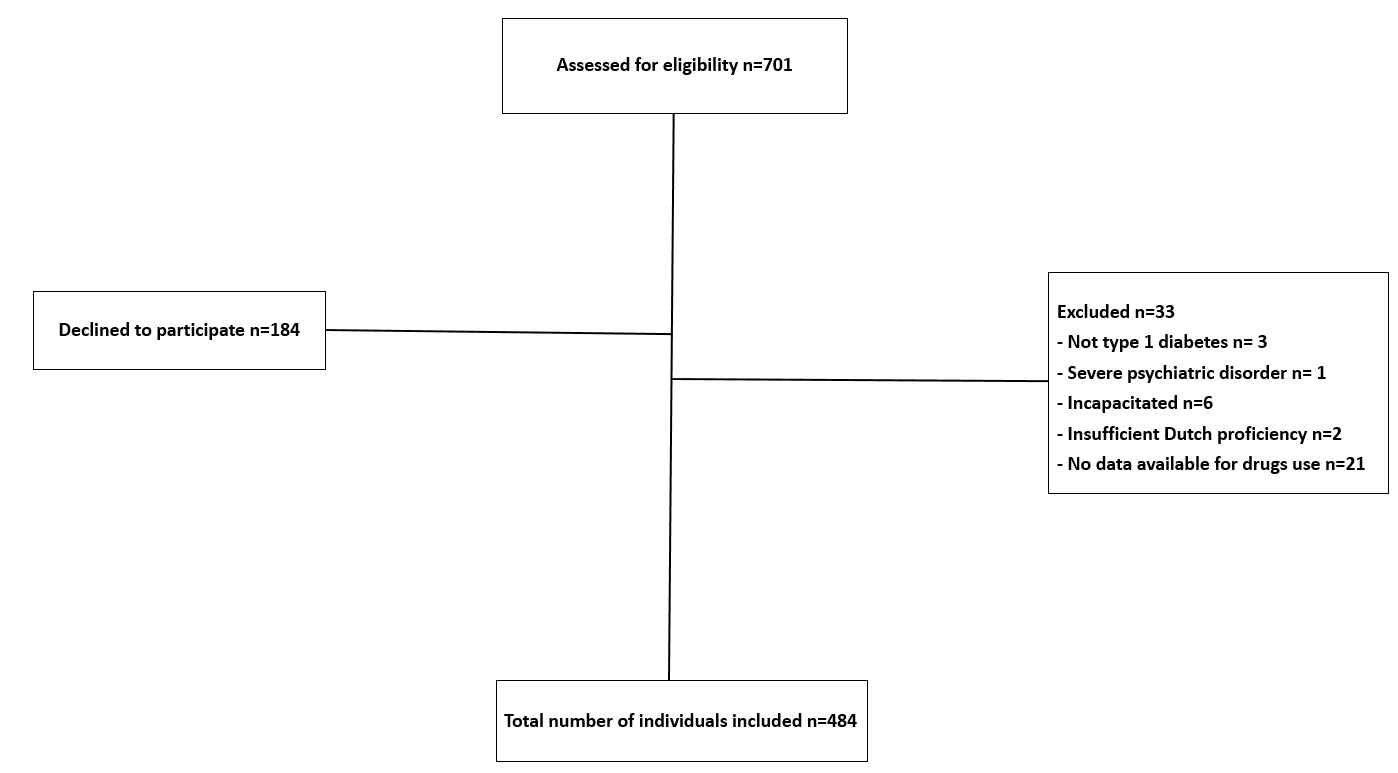

Supplement: Supplementary file 1 — Data S1. [file CPT-119-696-s001.docx]
